# Biogas Pollution and Mineral Deposits Formed on the Elements of Landfill Gas Engines

**DOI:** 10.3390/ma15072408

**Published:** 2022-03-24

**Authors:** Izabela Konkol, Jan Cebula, Lesław Świerczek, Magdalena Piechaczek-Wereszczyńska, Adam Cenian

**Affiliations:** 1 Physical Aspects of Ecoenergy Department, Institute of Fluid-Flow Machinery, Polish Academy of Sciences, Fiszera 14 Street, 80-231 Gdańsk, Poland; jcebula@imp.gda.pl (J.C.); lswierczek@imp.gda.pl (L.Ś.); cenian@imp.gda.pl (A.C.); 2 Institute of Environmental Engineering and Biotechnology, University of Opole, Kardynała Kominka 6, 6a Street, 45-032 Opole, Poland; mpiechaczek@uni.opole.pl

**Keywords:** landfill biogas, engine deposits, siloxane, hydrogen sulphide, lubricant pollution

## Abstract

Municipal landfills generate a significant amount of high-energy biogas, which can be used as a renewable gaseous fuel. However, it is necessary to improve the quality of this biogas due to the presence of various chemical compounds. The most common pollutants in landfill biogas include volatile compounds of silicon, sulphur, phosphorus and chlorine. The aforementioned elements, as well as other metals, were found both in the deposits and in the engine oil. The paper presents detailed characteristics of the solid residues formed in selected parts of gas engines powered by landfill biogas. Its elemental composition and morphology were investigated in order to determine the structure and influence of these deposits. In order to better understand the observed features, selected analyses were also conducted for biogas, engine oil and the condensate generated during biogas dewatering. It was found that the content of individual elements in samples collected from the same part of the gas engine but sourced from various landfills vary. The occurrence of elements in deposits, e.g., Mg, Zn, P and Cr, depends on the location of sampling sites and the type of engine. It was also observed that the deposits formed in parts that come into contact with both biogas and engine oil contain Ca or Zn, which can be related to biogas pollutants as well as different oil additives. The presence of Al, Fe, Cu, Cr, Sn or Pb in selected motor oil samples can be explained by the penetration of metallic abrasives, which confirms the abrasive properties of the formed deposits. The analysis of the characteristic deposits may contribute to the selection of an appropriate landfill biogas purification technology, thus reducing the operating costs of energy cogeneration systems. Finally, we highlight challenges for biogas purification processes and anticipate the direction of future work.

## 1. Introduction

Every year, the amount of generated waste increases, mainly due to industrial production and the increased consumption of goods. This is especially the case in highly developed countries. Depositing waste in landfills is still the most popular method of waste management in the world due to its low costs [1,2,3]. Landfills emit huge amounts of greenhouse gases, mainly methane (35–65%) and carbon dioxide (15–50%). On a global scale, municipal waste landfills are the third largest source of anthropogenic methane emissions (about 12%) [4,5,6,7,8,9].

In order to be considered as a renewable energy source, biogas should contain at least 60% of methane and have no organic and inorganic pollutants [10,11,12,13]. The high calorific value of landfill gas (about 11.9 to 19.8 MJ/m^3^) means that it can be used as a fuel in ICE (internal combustion engines) for electricity and heat production. Moreover, due to the constant generation of waste, landfill biogas can be considered to be an inexhaustible source of energy [14,15,16,17,18]. Its composition is closely related to the type of deposited waste and the type of anaerobic digestion processes. Due to the variety of chemical substances present in waste, landfill biogas, apart from methane and carbon dioxide, contains also H_2_O (0–5%), O_2_ (0–5%), H_2_ (0–3%) and CO (0–3%). Moreover, organic and inorganic compounds of sulphur, nitrogen, phosphorus and silicon are also present. The sulphur found in biogas is found in the form of volatile compounds: hydrogen sulphide, carbon oxysulphide, mercaptans, thioesters, etc. The nitrogen compounds include mainly ammonia, amines and nitrogen oxides.

Phosphorus is present mainly in the form of volatile phosphine. Volatile chlorine-containing compounds are present as aliphatic and aromatic chlorinated hydrocarbons, whereas organic silicon compounds exist in the form of silanes and siloxanes [19,20,21]. Siloxanes belong to the group of linear or cyclic compounds with repeating silica–oxygen atom sequences surrounded by methyl groups. These compounds are most often found in personal care consumer products (shampoos and cosmetics), antifoaming agents, detergents and polymeric silicone products (as precursors) [1,20,22,23,24]. Moreover, the polydimethylsiloxane polymers used in a wide range of industrial and domestic applications are dispersed in various environmental facilities [25]. Thus, the problem of biogas contamination with siloxanes is observed both in landfill biogas and biogas from sewage treatment plants. Substances generated by the decomposition of siloxane-containing products are present and concentrated in the water phase and, thus, are released and transferred to biogas due to their high vapor pressure and water solubility during anaerobic digestion [26,27,28,29,30]. Despite the frequent detection of siloxanes in the sewage sludge and produced biogas, their fate during the anaerobic digestion of the sludge has not been investigated so far [20,28].

**Figure 1:**
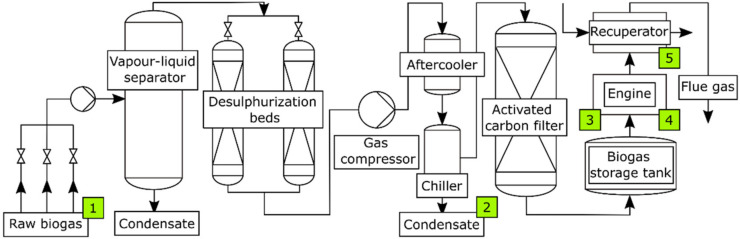
Biogas purification system: 1—biogas before purification, 2—condensate, 3—engine deposits, 4—oil, 5—recuperator deposit.

The implemented standard biogas purification setups—see, e.g., Figure 1, although effective in cleaning, e.g., sulphur compounds, are not efficient enough in removing all organic silicon compounds, which is shown in their presence in chiller condensate (see, e.g., [31] and Section 3.1) and engine deposits. However, the presence of siloxanes has a highly negative impact on biogas quality and its use. During combustion, silicone compounds transform into glassy microcrystalline silica, which damages various parts of the engine and reduces thermal conductivity.

Under normal engine operating conditions, a thin layer of oil forms on the surface of the cylinder liner, which serves to separate the liner from the piston ring. However, when using aggressive gases (including silicon compounds), the mineral structures that form during combustion can absorb the lubricant, thus reducing the amount available to ensure effective lubrication. Moreover, siloxanes are chemically converted into various forms of silicon compounds that can be highly abrasive. During operation, engine oil, which is an important structural element of a gas engine, changes its quality parameters, which is a natural and inevitable process. However, a lack of the appropriate thickness of the lubricating layer can lead to increased friction between the moving parts of the engine and can change the oil’s properties. This may contribute to the accelerated degradation of engine parts and more frequent wear of the engine components. Crankshaft and bearings are particularly sensitive, and hard particles can become trapped in their soft layers, leading to costly repair or even parts replacement. These particles can cause scratches and even cuts on the liner surface and wear on the piston rings. Deposits can also alter the geometry of the combustion chamber, increasing the release of carbon monoxide and formaldehyde. Detachable sediment fragments may cause the blocking of the cylinder liner, and the growing layers may inhibit heat conduction and the effective lubrication of cooperating engine components. Deposits typically accumulate in the combustion chamber on valves, valve seats, piston bottoms and cylinder walls [32,33,34,35,36].

When the organic silicon compounds enter the combustion chamber, they are oxidized, producing typical combustion components (e.g., carbon dioxide, silicone oxide and water) [34]. Silica or silicates, which are partially removed with the exhaust gas, can deposit on all engine components [34,35,37]. Deposits occur in the form of sediment layers several millimetres thick with a smooth or rough structure in various shades of grey, which are difficult to remove [23,33].

The organosilicon compounds in landfill biogas include D3 (hexamethylcyclotrisiloxane), D4 (octamethylcyclotetrasiloxane) and D5 (decamethylcyclopentasiloxane) [38]. The selected properties of commonly occurring organosilicon compounds in biogas were presented in our previous work [25]. As a result of the combustion process in the engine compartment, silicon dioxide (SiO_2(s)_) is formed [30,39]:

(1) $${\left({\left({\mathrm{CH}}_{3}\right)}_{2}\mathrm{SiO}\right)}_{4}+16O_{2}\to 4{\mathrm{SiO}}_{2}+8{\mathrm{CO}}_{2}+12H_{2}O$$

(2) $${\left({\left({\mathrm{CH}}_{3}\right)}_{2}\mathrm{SiO}\right)}_{5}+20O_{2}\to 5{\mathrm{SiO}}_{2}+10{\mathrm{CO}}_{2}+15H_{2}O$$

(3) $${\left({\left({\mathrm{CH}}_{3}\right)}_{2}\mathrm{SiO}\right)}_{6}+24O_{2}\to 6{\mathrm{SiO}}_{2}+12{\mathrm{CO}}_{2}+18H_{2}O$$

Additionally,

L2 (hexamethyldisiloxane) C_6_H_18_OSi_2_

$$C_{6}H_{18}{\mathrm{OSiO}}_{2}+12O_{2}\;\to 2{\mathrm{SiO}}_{2}+6{\mathrm{CO}}_{2}+9H_{2}O$$

L3 octamethyltrisiloxane C_8_H_24_O_2_Si_3_

L4 decamethyltetrasiloxane C_10_H_30_O_3_Si_4_

$$C_{8}H_{24}O_{2}{\mathrm{Si}}_{3}+16O_{2}\;\to 3{\mathrm{SiO}}_{2}+8{\mathrm{CO}}_{2}+12H_{2}O\;C_{10}H_{30}O_{3}{\mathrm{Si}}_{4}+20O_{2}\to 4{\mathrm{SiO}}_{2}+10{\mathrm{CO}}_{2}+15H_{2}O$$

$$C_{10}H_{30}O_{3}{\mathrm{Si}}_{4}+20O_{2}\;\to 4{\mathrm{SiO}}_{2}+10{\mathrm{CO}}_{2}+15H_{2}O\;C_{10}H_{30}O_{3}{\mathrm{Si}}_{4}+20O_{2}\to 4{\mathrm{SiO}}_{2}+10{\mathrm{CO}}_{2}+15H_{2}O$$

The silicone occurs in deposits mainly in the form of silicon dioxide and silicates. Their concentration depends on the quality of fuel introduced into the engine, as well as on the conditions in the combustion chamber. The study by Sevimoǧlu and Tansel [23] showed that the sum of the siloxane content in landfill biogas was around 9.5 ± 0.4 mg∙m^−3^, with the highest concentrations noted for D4 (5.0 ± 0.2 mg∙m^−3^), D5 (2.9 ± 0.1mg∙m^−3^) and L2 (1.6 ± 0.1 mg∙m^−3^). Depending on the type of biogas, silicon compounds can penetrate into an engine together with biogas or dust brought with the air, which can contain relatively large particles of mineral silicon compounds. Organosilicon compounds are also in antifoam agents introduced into the oil [33,40]. Therefore, the origin of silicon in the lubricant and deposits is difficult to determine; one should measure the silicon content twice, before and after changing the oil. The increase in the silicon content depends on the engine operating time, engine power and the volume of the replaced oil. Each engine producer determines the permissible amount of pollutants in biogas and in the exhaust gases [41,42].

In order to eliminate the negative impact of siloxanes on the condition of the engine, research is mainly carried out on biogas purification techniques. The basic techniques include:

- Activated carbon adsorption: the adsorption of siloxanes strongly depends on activated carbon parameters; however, overall silicon removal to concentrations below 0.1 mgSi·m^−3^ in non-continuous operations have been reported [43];
- Adsorption on silica gels [4,44];
- Molecular sieves [45];
- Absorption, the second major unit operation (physical), includes absorbents such as water, organic solvents or mineral soil [36], whereby chemical siloxanes are destroyed by strong bases and acids [45].

Above 99% of the siloxanes can be removed from the biogas stream by the technologies mentioned, reducing their concentrations to below 0.1 mg·m^–3^ [20,30]. Adsorbing materials (carbon-based materials) with a meso- and microporous structure seem to be the most technologically, technically and economically feasible [30]. Using an activated carbon bed, it was possible to significantly reduce the content of silicon compounds in the biogas; however, the resulting carbon deposits contained more sulphur than before the biogas purification process (104.6 ± 68.1 g∙kg^−1^). The reason for this was the release of the bed particles (containing sulphur) into a stream of purified biogas [46]. Around 10% of siloxanes can be removed during the high compression of the biogas in storage [34].

Shen and others [20] claimed that adsorption is relatively more suitable for siloxane removal than other technologies, but the occurrence of hydrogen sulphide interferes with the adsorption process. Moreover, the high cost of replacing the adsorption bed makes the process uneconomical. The methods commonly used to eliminate H_2_S also remove some siloxanes. Buch and Ingebrigston claim that an H_2_S adsorption tower (with limonite) reduces the concentration of siloxanes by 10% to 30% [34,47].

This paper aims to characterize biogas engine deposits, which can have a significant impact on the selection of the appropriate biogas purification technique and are helpful in estimating engine wear. The thorough monitoring of the chemical composition of deposits in gas engine parts can facilitate individual refinement of the landfill biogas treatment technology.

Research was also carried out to compare elemental composition and the morphology of deposits on different parts of the same engine. Microscopic and spectroscopic methods were used to observe the structure, morphology and composition of the deposits. The obtained results were compared with the existing literature data. For a better understanding of the processes taking place during the combustion of landfill biogas containing organosilicon substances, the biogas condensate that forms in the pipelines during biogas transport as well as engine oils were analysed.

## 2. Materials and Methods

The sites where samples were collected are shown in Figure 1.

**Figure 2:**
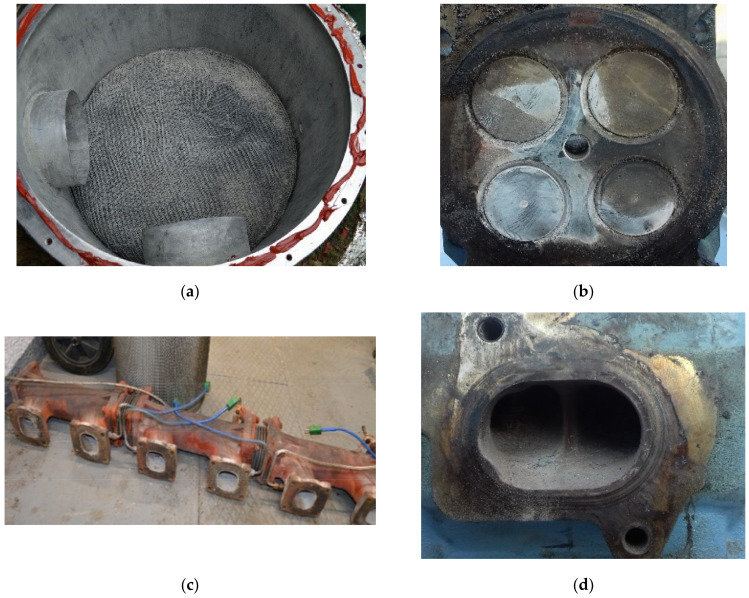
Deposits formed on different parts of engines: (**a**) fabric filter in front of the gas engine, (**b**) valves and (**c**,**d**) exhaust manifold.

**Figure 3:**
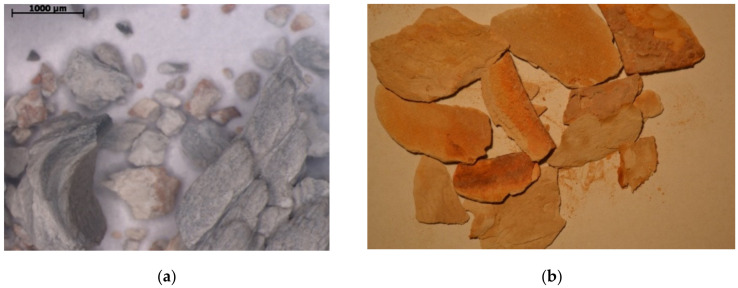
Deposits from: (**a**) head (optical microscope) and (**b**) recuperator.

Deposits were collected from different municipal solid waste landfills located in southern Poland (the landfills are labelled further in the text as: A, B, C, etc.). Samples were taken from various parts of gas engines: pistons, combustion chambers (valves, cylinder heads and spark plugs) and engine exhaust manifolds during technical service (Figure 2 and Figure 3a). Additionally, deposits formed in a recuperator were analysed (Figure 3b).

Samples were scraped from the parts on which they were mostly deposited using a sharp chrome–nickel steel knife, some of the deposits were loosely connected to the surface and could be easily sampled. After delivery to a laboratory, samples were ground using an agate mortar and stored under dry conditions.

The elemental composition of deposits on extreme surfaces and cross-sections was analysed with X-ray microanalysis (SEM-EDS) (SEM Microscope FEI Quanta FEG 250) using an X-ray spectrometer with energy dispersion (EDS). The voltage was kept at 15 kV. The phase composition and structure of deposits were analysed via X-ray diffraction (XRD) and X-ray fluorescence (XRF) spectrometry (Seifert-FPM XRD 7).

X-ray analyses of the crystalline phases of tested samples were performed on the basis of the interpretation of the diffraction pattern prepared with the use of the Seifert-FPM XRD 7 X-ray diffractometer. Characteristic CoKα radiation and an Fe filter were used. The diffractogram was made in the range of angles from 5° to 90°, which corresponds to the range of d_hkl_ interplanar lengths from 1.027 nm up to 0.1266 nm. The identification of compounds present in the sample was based on catalogues called the Powder Diffraction File, Search Manual (Hanawalt Method) and Inorganic JCPDS 1979. See the Powder Diffraction File, Sets 1–32, JCPDS 1974.

The deposits in the recuperator were studied in the case of relatively poorly cleaned landfill biogas, which is characterized by a high content of hydrogen sulphide. The samples were analysed for the content of dry residue using the gravimetric method, the content of sulphate ions was determined using ion chromatography and the content of iron was determined using atomic absorption spectrometry. For full characterization, condensate samples were also qualitatively analysed using gas chromatography coupled with a mass spectrometer (GC-MS) (Thermo Scientific, GC 2010, Waltham, MA, USA) to determine levels of organic compounds. Engine oils were analysed using inductively coupled plasma–mass spectrometry (ICP-MS) (Agilent 7700s, ICP-MS, Santa Clara, CA, USA).

The landfill biogas, powering gas engines, was also analysed. The hydrogen sulphide concentration was measured using a portable gas analyser (GA5000, Geotech, Manchester, UK). The analyser had ATEX II 2G Ex ib IIA T1 Gb (Ta= −10 °C to +50 °C), IECEx and CSA quality certifications and a UKAS ISO 17025 calibration certificate. The system allows the determination of hydrogen sulphide in the range of 0–5000 ppm. The calibration of the device was performed before experiments using calibration gases.

## 3. Results and Discussion

If gas engines located in landfills are powered by crude biogas, then their operation time shortens to around 100 h, even in the case of frequent exchange of lubricating oil. It was found that landfill crude biogas collected from a caption well could contain as much as 15,000 ppm of hydrogen sulphide. However, for the trouble-free operation of combined heat and power units, it is preferable that the H_2_S concentration in the biogas should be lower than 0.01 to 0.03% v/v, depending on the system considered [48]. Higher concentrations of hydrogen sulphide may cause the corrosion of the metal parts of the system and may contribute to air pollution by emitting, e.g., SO_2_ [49,50]. Moreover, the presence of hydrogen sulphide in biogas can lead to the formation of various minerals found in engine deposits.

### 3.1. Biogas Condensate

Landfill biogas contains significant amounts of steam as well as volatile carbon, silicon, sulphur, chlorine and phosphorus compounds. These pollutants occur also in agricultural biogas, as well as in biogas produced in municipal wastewater treatment plants. Volatile compounds, which are not decomposed under anaerobic conditions, diffuse into the biogas phase in the form of mists, vapours and gaseous solutions. It is worth mentioning that during the biogas temperature drop, the volatile organosilicon substances can condense. The amount and composition of the condensate depends on the cooling temperature. The lower temperature of the biogas, the more volatile compounds containing harmful substances for the engine will be condensed. Biogas condensate can also be produced in landfill gas collection systems [31].

**Figure 4:**
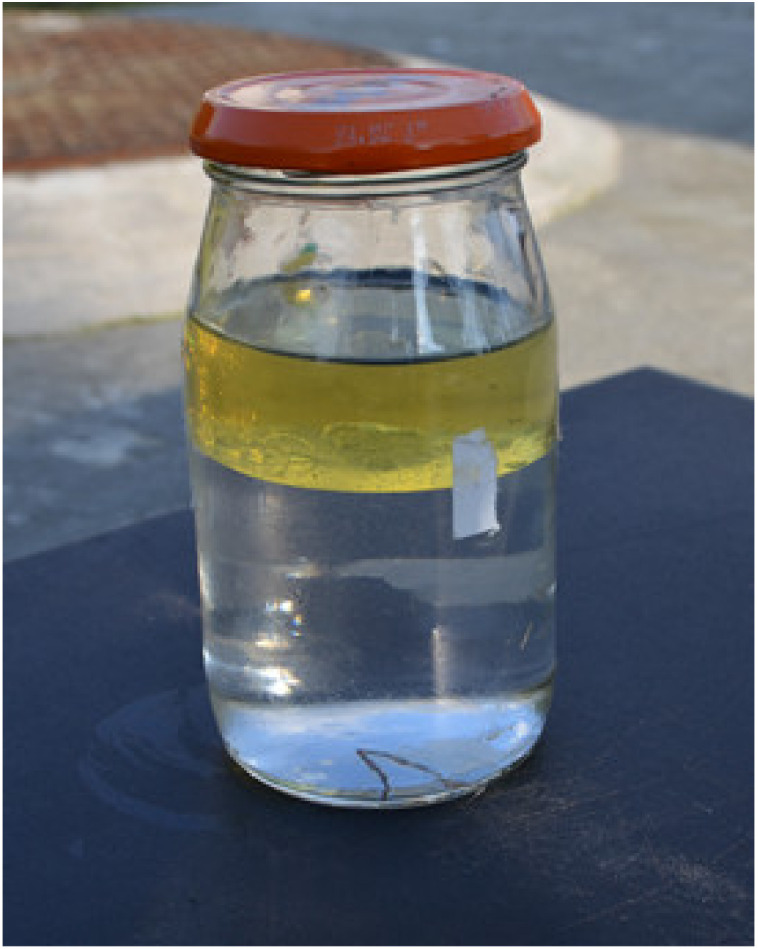
Condensate sample from landfill gas.

Biogas condensate is composed principally of water and organic compounds. Most organic compounds are not soluble in water, and the condensate separates into an aqueous phase and a floating hydrocarbon phase, which may comprise up to 5% of the liquid [31]. In most condensate samples, either in the water or organic phase, pollutant compounds can be found: benzene, toluene, 2-butanone (MEK), phenol, ethyl benzene, benzyl alcohol, bis (2-chloroisopropyl) ether, bis (2-ethyl-hexyl) phthalate, naphthalene, n-nitrosodimethylamine, 2,4-dimethylphenol and 4-methylphenol, and others [51]. Figure 4 presents a condensate sample, in which two immiscible organic and aqueous phases can be seen. The content of the insoluble matter in the condensate was 51 mg/g. The chemical analysis of the condensate proved the existence, next to sulphates, of small amounts of phosphate, fluoride, bromide, nitrate (V) and nitrate (III). On the other hand, the GC-MS qualitative analysis evidenced the presence of many organic compounds (see Table 1). These compounds belong to the group of aliphatic and aromatic hydrocarbons, which could have a detrimental impact on engine operations. As mentioned before, the compounds containing chlorine, fluorine and silicon atoms in their structure have the most critical effect. In the water phase of the condensate, silicone compounds were found 2.73% (w/w).

### 3.2. Solid Deposits

**Figure 5:**
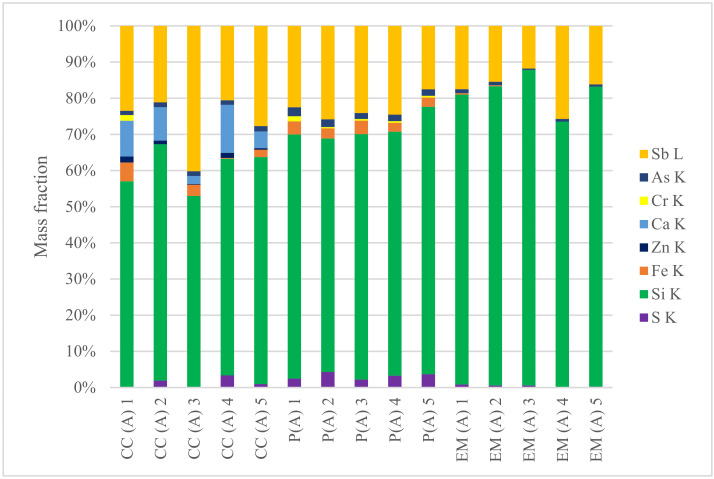
SEM-EDS quantitative microanalysis of deposits from extreme layers (CC—combustion chamber, P—piston, EM—exhaust manifold of engine from landfill A; numbers 1–5—different points of sampling; K and L—the atomic excited energy levels).

**Figure 6:**
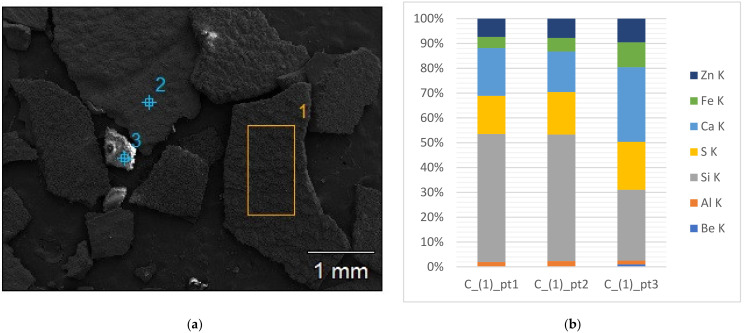
(**a**) SEM morphological characteristics at points (1, 2, 3) of deposit (piston, landfill C); (**b**) quantitative SEM-EDS microanalysis of the chemical composition of extreme layers of deposits sampled from the piston (landfill C), mass fraction (%).

The solid deposits from engines located in eight different landfills (denoted as A–H) were subjected to structural studies and elemental composition analysis. Elemental composition (in mass fractions) of deposits from different part of the gas engine (in the same landfill A) is presented in Figure 5. Figure 6a presents SEM photos of the external surface microstructures of exemplary piston deposits with the location of EDS sampling. In addition, Figure 6b shows the results of the SEM-EDS analysis of the piston deposit sample.

Even in the case of deposits taken from the same engine (landfill A), significant differences in their composition and characteristics were observed (Table 2). Mineral deposits from the exhaust manifold were characterized by a lower content of sulphur (0.02–0.84%) and arsenic (0.4–1.1%) compared to the sample taken from the piston face (2.19–4.3% for sulphur and 1.8–2.5% for arsenic) and the combustion chamber (0.94–3.4% for sulphur and 1.2–1.5% for arsenic). On the other hand, a higher content of silicon (81%) was observed in the exhaust manifold, and this was lower for the piston and combustion chamber at 68% and 60%, respectively. The second most abundant element was antimony (Figure 5). Its concentration changed gradually depending on the sampling site. The highest average content was noted for the combustion chamber (27%), and this was lower for the piston (23%) and the exhaust manifold (17%). In each deposit sample taken from individual parts of the gas engine, iron was detected. Its highest average content was found in the samples taken from the combustion chamber and piston (2.6 and 3.0%), and the lowest value (0.34%) was found in samples taken from the exhaust manifold. Calcium and zinc were observed only in the case of the combustion chamber at levels of 7.84% and 0.94%, respectively. It is worth mentioning that chromium was observed only in the deposit samples taken from the analysed gas engine (in landfill A).

**Figure 7:**
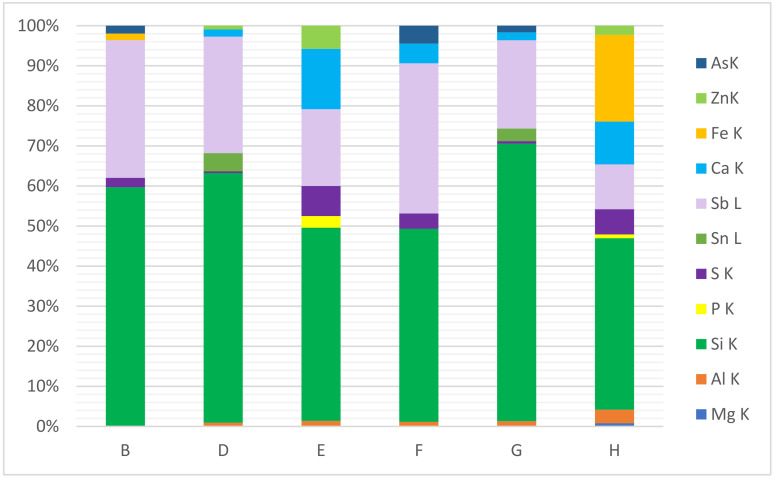
SEM-EDS quantitative microanalysis of extreme layers of deposits sampled from pistons (landfills B,D–H), mass fraction (%).

The piston, which is the main working element of any gas engine, is a place where mineral deposits are formed in abundance. Therefore, deposit samples collected from the piston faces of several gas engines were analysed. Figure 7 summarises the average results of the SEM-EDS analyses of deposits collected from six different piston faces of gas engines powered by landfill biogas.

It might be expected that the content of individual elements in deposits taken from the same part of the gas engine will be similar (Table 3). However, in the case of sulphur, the most significant (and largest) variations were observed. For the analysed piston face deposits, the content of sulphur, calcium and antimony ranged from 0.43 to 7.5%, 1.9 to 15% and 19 to 37%, respectively. Significant differences were also observed in the silicon content (48–69%); moreover, content of this element was on a comparable level for piston deposits sampled from landfills E, F and H (43–48%) and for landfills A, B, D and G (62–69%). Content variations were also observed in the case of iron (1.7–22%), and this element was observed only in piston deposits from three landfills: A, B and H. In these deposits, aluminium (the highest for landfill H at 3.4%), arsenic (the highest at 4.5% for landfill F) and zinc (the highest at 5.8% for landfill E) were also observed. The occurrence of the above-mentioned elements, as well as magnesium, tin, phosphorus and chromium, depends on the location of the gas collection site and the type of engine. Moreover, similar variations were observed in deposits from gas engines powered by biogas from a waste water treatment plant [25].

**Figure 8:**
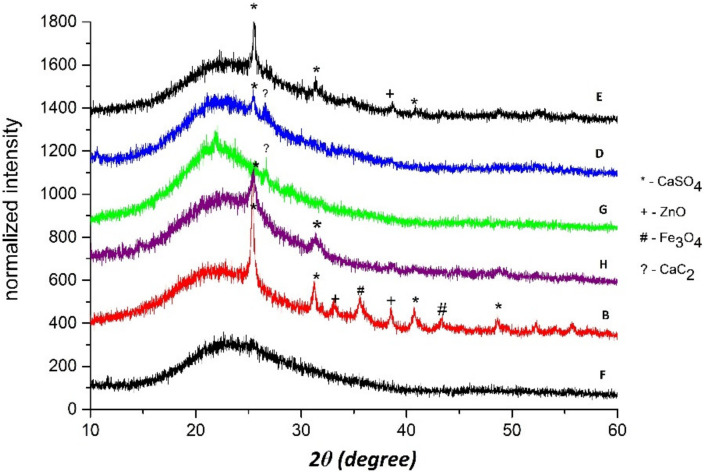
XRD analysis of piston deposits from engines located in landfills B, D–H.

An XRD analysis of piston deposits from engines located in landfills B,D–H (Figure 8) confirms the presence of compounds such as CaSO_4_, ZnO, Fe_3_O_4_ and CaC_2_. CaSO_4_ was found in the deposits from most landfills, including B, D, E and H, reflecting the higher concentration of hydrogen sulphur in the fuel. The ZnO oxide was found in the deposits from landfills B and E, and CaC_2_ was found in the deposits from landfills D and G (probably due to some problems with combustion), whereas Fe_3_O_4_ was found only in the deposit from landfill B. As was previously mentioned, Zn and Ca can be derived from engine oil, whereas Fe results from processes inside the cylinders [33].

In all the investigated deposits taken from gas engines, both sulphur and silicone were detected (determined). As mentioned earlier, this may be related to the combustion of biogas contaminated with siloxanes and hydrogen sulphide. Considering the variation of deposit compositions on engine components, it can be concluded that the temperature differences of individual engine parts and the cooling of exhaust gases may have a strong impact on the elemental content of deposits. The highest amount of silica was noticed in the exhaust manifold, where the exhaust gas temperature is lower, which causes the silica to condense more intensively. It is worth mentioning that Storey et al. [52] observed both amorphous and crystalline phases of silica in gas engines. Crystalline phases are caused by the slow cooling of the gas phase silica and occurred on the hottest surfaces of the engine, such as the piston crown. Amorphous silica (glass) is formed from the rapid cooling of the gases, typically near the cylinder walls and on the head of the combustion chamber. A higher exhaust temperature corresponds to a leaner burning condition, and this was also evidenced by the lack of hydrocarbon deposits on the cylinder head surfaces. For higher and lower exhaust temperatures, silica build-up was visible on the exhaust gas valve, spark plug and the exhaust manifold. The build-up was more evident for cylinders with lower exhaust temperature, and this may be related to increased condensation [52].

The elemental composition of deposits on pistons can be influenced by many factors. The temperature and exhaust gas flowrate, as well as the structure itself, may contribute to significant differences in the composition of the formed deposits. However, the silica formed in the combustion chamber can contribute to the increased friction of individual engine components. This can cause significant wear of the moving parts with the simultaneous release of elements from the alloys from which, e.g., bushings or bearings are made. Thus, deposits formed in the combustion chamber often have a higher arsenic and antimony content than deposits formed in the exhaust manifold [53]. However, due to the lack of more extensive research on the formation and deposition of the described compounds, it is difficult to clearly define the whole phenomenon.

### 3.3. Engine Oil

Table 4 presents the elemental composition of engine oils sampled from four landfill gas engines. The presence of elements such as zinc and calcium may be related to the impurities present in biogas and the chemical composition of the lubricating oil. It is highly probable that aluminium originates from the metallic layer of the engine piston face (Al and Fe alloy) [53] and other gas engine parts, such as bearings, the cylinder liner and engine blocks [54]. The presence of this metal can also be explained by the penetration into the motor oil of metallic abrasives containing Al, and also Fe, Cu, Cr, Sn or Pb [33,46].

The deposits on engine components that come into contact with both biogas and lubricating oil (e.g., pistons and the whole combustion chamber) will contain both elements related to biogas pollutants and different oil additives. For this reason, elements belonging to the oil additives, e.g., Ca or Zn, were found. Calcium and magnesium have anti-wear and antioxidant properties, and they act as a corrosion inhibitor and a dispersant. Phosphorus added to oils operating under extreme pressures shows similar properties. Zn used as an antioxidant and corrosion inhibitor has also anti-wear properties [53]. The presence of sulphur compounds, on the other hand, may result from burning biogas containing hydrogen sulphide [33,55]. The study by Sevimoǧlu and Tansel [23] showed that deposits on the engine heads combusting landfill biogas can contain a high content of silicon (149,400 ± 89,900 mg∙kg^−1^), calcium (70.84 ± 17.75 g∙kg^−1^), sulphur (42.5 ± 11.5 g∙kg^−1^) and zinc (0.022 ± 0.007 g∙kg^−1^).

It should be noted that the composition and morphology of the mineral deposits produced in engines powered by landfill biogas are different from the deposits formed during the combustion of agricultural biogas. For this reason, both types of gaseous fuels (landfill and agriculture biogas) should be pre-treated (cleaned) in different setups, taking into account the analysis of biogas pollutants and deposits formed, e.g., on engine heads and pistons.

Taking into account the heterogeneity of the tested samples in terms of chemical characteristics, it can be argued that the different morphological composition results from the variable composition of biogas from different landfills and variable engine operation conditions [33]. The characteristics and working environment of a gas engine are influenced by many factors, such as the chemical composition of the fuel as well as the composition of the engine oil, the frequency of its refilling and replacement, the engine operation stage, the scope and frequency of repairs and the method of servicing the engine.

## 4. Conclusions

- For the proper functioning of a landfill biogas plant, it is necessary to properly design and monitor/analyse the work of biogas cleaning devices. Otherwise, the engine will seize quickly due to deteriorating lubricating properties of the oil.
- Deposits are formed not only in the combustion chamber, but also in other components of the engine. The chemical composition of deposits taken from the combustion chamber or the exhaust manifold of the engine differ substantially due to deposit fractionation. Volatile and dusty compounds are either blown into the chimney or washed into the lubricating oil. On the other hand, glassy compounds crystallize on the colder parts of the engine.
- The monitoring of both the biogas and the solid deposits (mainly the content of silicon and sulphur) characteristics is necessary in order to select the appropriate method of biogas purification and the time interval between the technical inspections of the gas engine and oil change.
- In order to limit the development of sediments with the predominant content of hard crystalline silicon compounds, the use of properly selected enriching additives for engine oils, appropriate for various stages of engine operation, should be considered. The presented view may be a premise for further research on the formation of harmful deposits in gas engines.
- A microanalysis of the chemical composition of the sediments showed, inter alia, the presence of phosphorus compounds. This is a lubricating oil-refining additive. Compounds of this element can also penetrate into landfill gas (e.g., phosphine), resulting in exceptional toxicity.
- The morphological composition of wastes in various landfills varies significantly, which influences the composition of the biogas produced. Therefore, the development of a proper biogas purification technology will require extensive studies and an individual approach for any landfill.
- Future studies will focus on the speciation analysis of deposits that appear on various parts of the gas engine, as well as contaminations of lubricant oil; this can allow the development of recipes for new lubricating oils that are resistant to contaminants in biogas. It could also enable the optimization of the engine operating conditions in order to reduce the amount of pollution emitted from the gas engines.

There is a need for further studies, including a thorough investigation of the contaminants in lubricant oils, the deposits on fixed and moving engine parts, the deposits on exhaust pipes and the content of exhaust gases.

## Figures and Tables

**Table 1:** Organic compounds detected by GC-MS in biogas condensate.

No	Name	No	Name
Organic phase			
1	Chlorobenzene	14	Heptanol
2	Cycloheptane	15	Isopulegol
3	Cyclohexanol	16	Methylene-butanediol
4	Cyclohexanone	17	Methylheptane
5	Cyclohexasiloxane	18	Methylpentane
6	Cyclopentasiloxane	19	Methyl propyl pentanol
7	Dichloroethane	20	Pentane
8	Dichloroethylene	21	Propylbenzene
9	Dichlorofluoromethane	22	Tetrachloroethene
10	Ethylmethylcyclohexane	23	Tetradecane
11	Ethylmethylcyclopentane	24	Tetramethylbutane
12	Heptadecane	25	Tetramethylpentane
13	Heptadecane		
Water phase			
1	Toluene		
2	Cyclopentasiloxane		
3	Cyclohexanone		
4	Cyclohexanol		
5	Cyclohexasiloxane		

**Table 2:** Chemical composition of extreme layers of deposits (landfills A–C), weight share (%).

Gas Engine Element		Combustion Chamber (A)					Piston (A)					Piston (B)					Exhaust Manifold (A)				
**Sample**		**1**	**2**	**3**	**4**	**5**	**1**	**2**	**3**	**4**	**5**	**1**	**2**	**3**	**4**	**5**	**1**	**2**	**3**	**4**	**5**
**Element mass (%)**	Be K	-	-	-	-	-	-	-	-	-	-	-	-	-	-	-	-	-	-	-	-
	Al K	-	-	-	-	-	-	-	-	-	-	-	-	-	-	-	-	-	-	-	-
	S K	-	1.92	-	3.37	0.94	2.48	4.30	2.19	3.27	3.70	1.39	-	6.16	1.10	0.76	0.84	0.56	0.57	0.02	0.27
	Si K	57.03	65.35	52.98	59.89	62.79	67.51	64.56	67.88	67.47	73.92	68.20	50.62	45.14	68.95	66.98	80.15	82.76	87.24	73.50	82.94
	Fe K	5.20	-	3.11	0.22	2.03	3.60	2.80	3.72	2.45	2.54	-	1.01	5.95	0.55	0.78	0.40	0.27	-	-	-
	Zn K	1.66	1.02	0.20	1.40	0.42	-	-	-	-	-	-	-	-	-	-	-	-	-	-	-
	Ca K	9.90	9.23	2.19	13.29	4.62	-	-	-	-	-	-	-	-	-	-	-	-	-	-	-
	Cr K	1.56	-	-	-	-	1.44	0.37	0.46	0.45	0.51	-	-	-	-	-	-	-	-	-	-
	As K	1.17	1.35	1.33	1.24	1.50	2.48	2.15	1.66	1.82	1.78	2.19	1.64	1.72	1.82	2.46	1.10	0.94	0.40	0.75	0.62
	Sb L	23.48	21.14	40.19	20.59	27.70	22.50	25.82	24.09	24.53	17.56	28.22	46.73	41.03	27.58	29.02	17.51	15.47	11.79	25.73	16.17

**Table 3:** Chemical composition of extreme layers of deposits from piston from different landfills (landfill C, and average composition for landfills D–H), weight share (%).

Sample		C_(1)_pt1	C_(1)_pt2	C_(1)_pt3	D	E	F	G	H
**Elemental weight (%)**	Be K	-	-	0.99	-	-	-	-	-
	Al K	1.92	2.33	1.5	-	-	-	-	-
	Mg K	-	-	-	-	-	-	-	0.78
	Al K	-	-	-	0.92	1.38	1.15	1.30	3.40
	Si K	51.6	50.97	28.57	62.31	48.23	48.17	69.27	42.79
	P K	-	-	-	-	2.89	-	-	0.97
	S K	15.38	17.12	19.3	0.43	7.52	3.84	0.64	6.27
	Sn L	-	-	-	4.55	-	-	3.14	-
	Sb L	-	-	-	29.07	19.16	37.46	22.05	11.19
	Ca K	19.23	16.34	30.08	1.80	15.06	4.93	1.95	10.67
	Fe K	4.49	5.45	10.03	-	-	-	-	21.67
	Zn K	7.37	7.78	9.53	0.92	5.76	-	-	2.26
	As K	-	-	-	-	-	4.45	1.66	-

**Table 4:** Engine oils analyses.

Oil Engine				
Oil Hours	A	B	C	D
	600	325	-	-
Content (ppm)				
Fe	13	3	12	8
Al	1	3	13	7
Si	12	211	-	-
Ca	3195	1631	1600	1700
Mg	12	5	-	-
B	1	0	23	22
Zn	3	349	411	465
P	7	278	-	-
S	6439	3427	-	-

## Data Availability

The data presented in this study are available on request from the corresponding author.
